# Enhancing SELD Performance: The Role of Data Augmentation Techniques in Spatial Sound Analysis

**DOI:** 10.3390/s26113466

**Published:** 2026-05-31

**Authors:** Christian Santamaria, Felipe Grijalva, Karen Rosero, José Vega-Sánchez, Nathaly Orozco Garzón, Henry Carvajal Mora

**Affiliations:** 1 Colegio de Ciencias e Ingenierías “El Politécnico”, Universidad San Francisco de Quito USFQ, Diego de Robles S/N, Quito 170157, Ecuador; csantamarial@estud.usfq.edu.ec (C.S.); dvega@usfq.edu.ec (J.V.-S.); hcarvajal@usfq.edu.ec (H.C.M.); 2 Language Technologies Institute, Carnegie Mellon University, Pittsburgh, PA 15213, USA; kroseroj@andrews.cmu.edu; 3 Faculty of Engineering and Applied Sciences, Networking and Telecommunications Engineering, ETEL Research Group, Universidad de Las Américas (UDLA), Quito 170503, Ecuador; nathaly.orozco@udla.edu.ec

**Keywords:** Acoustic Signal Processing, Acoustic Scene Analysis, Sound Event Localization and Detection, data augmentation, deep learning, spatial sound

## Abstract

Sound Event Localization and Detection (SELD) integrates Sound Event Detection (SED) and Direction-of-Arrival Estimation (DOAE) to recognize and localize sound events in various applications, including urban sound sensing, wildlife monitoring, and home surveillance. Recently, advancements in machine learning, particularly deep learning techniques, have demonstrated remarkable success in improving SELD performance. However, training deep learning models for SELD is challenged by the limited availability of high-quality spatial audio data, which is essential for accurate model generalization. This paper explores the effectiveness of data augmentation techniques in overcoming this limitation. We evaluate the impact of Frequency Shift (FS), Random Cutout (RC), and Channel Swapping (CS) on SELD performance using a comprehensive set of experiments. Our findings indicate that all tested augmentation combinations except FS alone significantly improve SELD performance, reducing the SELD error by approximately 8% compared to no augmentation. The differences among effective combinations are not statistically significant, suggesting that the decision to augment is more impactful than the specific combination chosen. This work highlights the critical role of data augmentation in enhancing SELD systems and suggests future research directions, including testing these techniques with different model architectures and exploring additional augmentation methods.

## 1. Introduction

Sound Event Localization and Detection (SELD) merges Sound Event Detection (SED) with Direction-of-Arrival (DOA) estimation, enabling the identification of sound classes and the estimation of their spatial attributes. SELD is crucial for applications such as urban sound sensing [1], wildlife monitoring [2], and home surveillance [3]. By leveraging multichannel audio from microphone arrays, SELD enables precise localization of sound sources in complex environments [4,5,6].

A key challenge in SELD is the limited availability of high-quality spatial audio data, which is difficult to collect due to the need for specialized equipment and controlled conditions [7]. This data scarcity hampers the training of deep neural networks, which require large datasets to generalize effectively. Consequently, data augmentation (DA) has emerged as a crucial strategy for enhancing SELD models by generating diverse training samples.

Table 1 shows the main approaches in DA techniques to improve SELD performance. Wang et al. [8] introduced a four-stage strategy integrating Audio Channel Swapping (ACS), Multi-Channel Simulation (MCS), Time-Domain Mixing (TDM), and Time-Frequency Masking (TFM), effectively addressing data sparsity. Similarly, Zhang et al. [9] incorporated ACS and TFM into a CNN-Conformer architecture, while Ronchini et al. [10] employed Time-Stretching (TS), Pitch Shifting (PS), and Channel Rotation (CR) in a Convolutional Recurrent Neural Network (CRNN), identifying CR as the most effective.

Additional contributions include SpecAugment-based techniques by Zhang J. et al. [11] and the use of Spatial cue-Augmented Log-SpectrogrAm (SALSA) features alongside Random Cutout (RC), Channel Swapping (CS), and Frequency Shift (FS) by Nguyen et al. [12]. Furthermore, in [13], DA techniques such as Gain Augmentation, Noise Augmentation, and SpecAugment were successfully applied to enhance transformer-based SELD models. These techniques introduced controlled variations in amplitude, added adaptive colored noise, and leveraged time–frequency masking to improve model robustness, reinforcing the importance of augmentation strategies for SELD performance.

Building on these insights, this study investigates the impact of various DA techniques specifically designed for spatial audio. The proposed approach focuses on three key augmentation methods: (1) traditional per-channel audio augmentations such as Gain and Noise augmentation, (2) SpecAugment applied to feature encoder outputs for time and frequency masking, and (3) Sound Field Rotation using Channel Swapping to manipulate the spatial representation in First-order Ambisonics (FOA) format audio. These techniques are applied during the fine-tuning phase to address dataset limitations and improve SELD model generalization. The effectiveness of these augmentations is systematically evaluated, demonstrating their contribution to handling overlapping events and mitigating data scarcity challenges. The findings presented in this work contribute to advancing SELD methodologies by leveraging DA to enhance spatial audio learning and model robustness.

## 2. Methodology

In this section, we describe the features used for model training, along with a detailed explanation of the data augmentation techniques applied. Next, we break down the architecture of the deep learning model employed, SELDnet. Finally, we report the dataset used, the training hyperparameters, and the hardware utilized for experimentation.

### 2.1. Audio Features for SELD

The study employed multichannel log-mel and log-linear spectrograms with intensity vectors (IV), referred to as MelSpecIV and LinSpecIV respectively. Specifically, IV provides directional information by calculating intensity differences between microphone array channels. Mel spectrograms, based on human auditory perception, emphasize low frequencies and capture relevant signal information. Linear spectrograms maintain frequency resolution across the spectrum, preserving precise directional information. FOA includes four channels of signals, i.e., omni-directional channel *w*, and three directional channels *x*, *y*, and *z*. Log-mel spectrograms are computed from the short-time Fourier transform (STFT) spectrograms of four-channel signals, and IVs are computed from cross-correlation of *w* with *x*, *y* and *z* in log-mel space [14]. These features improve the detection and localization of sound events in noisy and reverberant environments.

Additionally, SALSA features, a novel technique for SELD, were employed to map the signal power to the source DOA in a spectral–temporal domain. In particular, SALSA consists of multi-channel linear-frequency log-magnitude spectrograms stacked with a normalized version of the principal eigenvector of the spatial covariance matrix at each time–frequency (TF) bin of the spectrograms [12]. This feature has proven effective for both FOA and microphone array (MIC) formats, improving sound event detection and localization in various environments.

All spectral features were computed using the librosa Python library (version 0.8). Specifically, STFT computation was performed via librosa.stft with an FFT size of 512 and a hop length of 300 samples, while mel-frequency filterbanks were obtained through librosa.filters.mel with 128 mel bands ($f_{\min}=50$ Hz). Log-power spectrograms were computed using librosa.power_to_db. Audio files were loaded at the native sampling rate of 24 kHz via librosa.load.

### 2.2. Data Augmentation Techniques

Three data augmentation techniques were utilized in our study, namely, FS, RC, and CS, along with all possible combinations applied to all features. The choice and combination of these techniques aim to enhance the robustness and generalization capability of the SELD model.

#### 2.2.1. Frequency Shift

This method alters the frequencies of the input audio features by shifting the pre-extracted spectrograms along the frequency axis. Specifically, input features are randomly shifted by up to Δf=10 frequency bins using reflect-mode boundary padding. For MelSpecIV features, these bins correspond to mel-frequency bands; for LinSpecIV and SALSA features, they correspond to linear frequency bins. Since the operation is applied to magnitude spectrograms rather than complex-valued STFT representations, no phase reconstruction is required. This helps simulate different pitch variations and can make the model more robust to such variations. The shift magnitude is sampled from a discrete uniform distribution Δf∼U{1,10}, and the direction (toward higher or lower frequencies) is chosen with equal probability. When applied, reflect-mode padding is used to fill the vacated bins, preserving continuity at the spectral boundaries. This technique is applied independently to each training sample with a probability p=0.5.

#### 2.2.2. Random Cutout

This technique involves replacing a randomly selected region of the input with random values [15] or applying time–frequency masking, as done in SpecAugment [16]. By obscuring parts of the input, this method encourages the model to rely on contextual cues from the remaining signal, enhancing its ability to generalize and handle occlusions or missing information. In our implementation, Random Cutout is realized as a composite strategy that randomly selects one of three masking variants with equal probability at each application: (i) *rectangular cutout*, which masks a contiguous rectangular region whose area is sampled uniformly between 2% and 30% of the spectrogram area, with an aspect ratio drawn to match the time–frequency proportions of the input; (ii) *SpecAugment*, which applies one time stripe (up to 15% of the total frames) and one frequency stripe (up to 20% of the frequency bins), following [16]; and (iii) *cutout holes*, which places 8 small rectangular holes of 8×8 bins at random positions. For all three variants, the masked regions in the spectral channels are filled with values drawn uniformly between the minimum and maximum of the input spectrogram, while the intensity vector channels (last 3 channels in the 7-channel FOA representation) are filled with zeros. The composite cutout is applied to each training sample with a probability p=0.5.

#### 2.2.3. Channel Swapping

This consists of permuting the channels of the input features while adjusting the corresponding ground truth labels [17]. Beyond simulating different microphone placements, channel swapping effectively alters the spatial characteristics of the sound, mimicking variations in source localization within a 3D space. For instance, in a four-microphone array, reordering the channels replicates different acoustic environments, helping the model become more robust to changes in microphone configuration and spatial positioning. Channel Swapping is applied independently to each training sample with a probability p=0.5.

Formally, for FOA-format features organized as $\left[W,Y,Z,X,{\mathrm{IV}}_{y},{\mathrm{IV}}_{z},{\mathrm{IV}}_{x}\right]$ (7 channels) and class-wise DOA labels in Cartesian coordinates $y_{\mathrm{doa}}=\left[x_{1}\ldots x_{K},y_{1}\ldots y_{K},z_{1}\ldots z_{K}\right]$ for *K* classes, four independent binary random variables $m_{0},m_{1},m_{2},m_{3}\in \left\{0,1\right\}$ are sampled. If $m_{0}=1$, the X and Y directional channels are swapped in the features, and the *x*- and *y*-coordinates are swapped in the DOA labels. If $m_{1}=1$, the X intensity vector is negated and the *x*-coordinates in labels are negated, similarly for $m_{2}$ (Y axis) and $m_{3}$ (Z axis). These operations correspond to reflections across the coordinate planes and axis permutations in 3D Cartesian space, yielding up to $2^{4}=16$ valid spatial configurations while preserving full label consistency.

By applying the above techniques in all possible combinations, we aim to create a comprehensive augmentation strategy that covers a wide range of real-world scenarios. This combined approach ensures that the model learns to generalize well across various types of distortions and variations present in audio signals.

### 2.3. Deep Learning Model for SELD

**Figure 1:**
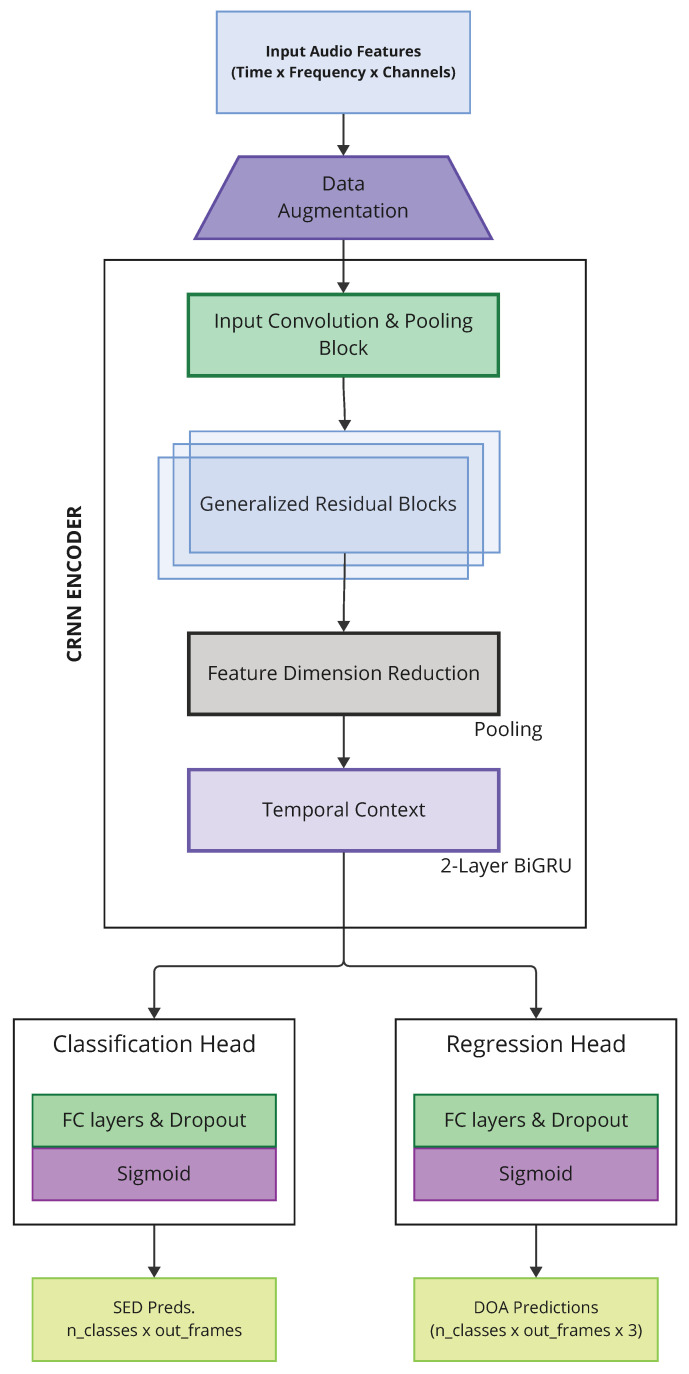
Block diagram of the SELD system [12].

The network architecture utilized builds upon the original SALSA proposal, as illustrated in Figure 1. Here, a CRNN comprises a convolutional structure based on ResNet22, as proposed in the Pretrained Audio Neural Networks (PANN) framework [18]. This architecture consists of an initial convolutional block followed by four residual blocks with progressively increasing filter sizes (64, 128, 256, 512), followed by a two-layer BiGRU, and fully connected (FC) output layers. The number of input channels in the first convolutional layer is adjusted to the number of channels of each feature under study. The SED branch is formulated as a multi-label and multi-class classification, while the DOA estimation branch is formulated as a three-dimensional Cartesian regression [19].

### 2.4. Datasets for SELD

The selected dataset for this work is TAU-NIGENS Spatial Sound Events 2021 [20], used in the DCASE 2021 Challenge, which comprises recordings of spatial sound scenes with events in various acoustic spaces. In particular, it includes 600 one-minute recordings with metadata that provides sound classification labels and corresponding Direction-of-Arrival (DOA) annotations for development and 200 recordings without metadata for evaluation. Each sound event is associated with a static DOA or a trajectory of DOAs for moving events, along with start and end times. Recordings are available in MIC and FOA formats, with a sampling frequency of 24 kilohertz (kHz) and 12 predictable output classes.

### 2.5. Experimental Setup

We adopt the hyperparameter configuration of Nguyen et al. [12]: a sampling rate of 24 Hz and a window length of 512 samples with a hop length of 300 samples were used for audio processing, resulting in an input frame rate of 80 frames per second (fps). The model temporally downsampled the input by a factor of 16, and the outputs were temporally upsampled by a factor of 2 to match the label frame rate of 10 fps. This downsampling–upsampling configuration maps the input rate to the label annotation rate required by the DCASE evaluation protocol (80/16×2=10 fps), and follows the original SALSA architecture; it was held constant across all experimental conditions to isolate the effect of data augmentation. To optimize training time, frequency bands above 9 kHz were compressed by a factor of 8, reducing the frequency dimension to 200. This compression preserves the spectral range where most sound events in the dataset concentrate their energy, while substantially reducing computational cost. This parameter also follows the SALSA baseline and was fixed across all conditions. Training was conducted with 8-s audio chunks, using the Adam optimizer with an initial learning rate of $3\times 10^{-4}$, which was reduced to $10^{-4}$ over the last 15 epochs of a total of 50 epochs. Finally, a threshold of 0.3 was used to binarize predictions in sound event detection. For the implementation, PyTorch version 2.9.0 and Lightning libraries were primarily used for model management, data handling, and the integration of data augmentation techniques. The experiments were conducted on an NVIDIA A100 GPU, equipped with 80 GB of dedicated memory.

### 2.6. Evaluation Metrics

The evaluation metrics used in the SELD task are crucial for assessing the performance of systems developed to detect and localize sound events in a spatial environment. We adopted the metrics presented in [21], i.e., *Localization Error (LE)*, *Localization Recall (LR)*, *Detection Error Rate (ER)*, and *F1-Score*. In this section, we provide an in-depth explanation of each metric.

#### 2.6.1. Localization Error (LE)

The *LE* quantifies the accuracy with which a SELD system can estimate the DOA of a sound event. This metric is particularly important in tasks where the spatial location of the event is as critical as its detection. The LE is typically expressed in degrees, with lower values indicating more accurate localization.

#### 2.6.2. Localization Recall (LR)

The *LR* evaluates the system’s ability to correctly detect and localize sound events, assessing the percentage of events that are accurately localized within an acceptable localization error threshold. The *LR* is expressed as a percentage, with higher values indicating that the system is effective at both detecting and localizing sound events accurately.

#### 2.6.3. Detection Error Rate (ER)

The *ER* measures the system’s ability to detect the occurrence of sound events, irrespective of their localization. It focuses on the error rate in detection, where an error can be a false alarm (detecting a non-existent event) or a miss (failing to detect an existing event). The ER ranges from 0 to 1, where 0 indicates perfect detection, and 1 indicates complete detection failure.

#### 2.6.4. F1-Score

The *F1-Score* is a metric that combines precision (accuracy in detecting events) and recall (ability to find all events) into a single value. It is particularly useful when there is a need to balance precision and recall. The F1-Score ranges from 0 to 1, where a value of 1 indicates perfect precision and recall, and 0 indicates complete failure in either precision or recall. Mathematically speaking, the *F1*-score is calculated as by

(1) $$F1=\frac{2\sum_{k=1}^{K}TP\left(k\right)}{2\sum_{k=1}^{K}TP\left(k\right)+\sum_{k=1}^{K}FP\left(k\right)+\sum_{k=1}^{K}FN\left(k\right)},$$

where true positives TP(k), false positives FP(k), and false negatives FN(k) are evaluated in the *k*th one-second segment of the sample [22].

#### 2.6.5. Relationship Between Metrics

In the context of SELD, *LE*, *LR*, *ER*, and *F1* are used collectively to evaluate the system’s performance, as a balance between accurate event detection and precise localization is crucial. Each metric offers a distinct perspective, and together they provide a more comprehensive and robust assessment of the model. This holistic approach led to the development of the SELD error metric [23], which integrates all the metrics as expressed by

(2) $$E_{\mathrm{SELD}}=\frac{1}{4}\left[{\mathrm{ER}}_{\leq 20^{\circ }}+\left(1-{F1}_{\leq 20^{\circ }}\right)+\frac{{\mathrm{LE}}_{\mathrm{CD}}}{180^{\circ }}+\left(1-{\mathrm{LR}}_{\mathrm{CD}}\right)\right].$$

The subscript *CD* stands for *Class-Dependent*; this indicates that metrics such as the localization error ($LE_{CD}$) and the localization recall ($LR_{CD}$) are calculated while considering the specific differences between sound event classes. This provides a more detailed evaluation of the system’s performance for each class.

## 3. Experiments and Results

Since the model does not process raw audio, the first step was to extract audio features—MelSpecIV, LinSpecIV, and SALSA—from the entire training dataset. These features were stored in separate directories for later use. After the feature extraction process, the development dataset, comprising six folds of 100 recordings each, was split as follows: the sixth fold was reserved as a held-out evaluation set, while the remaining five folds were used in 5-fold cross-validation. In each iteration, four folds (400 recordings) served as training data and one fold (100 recordings) was used for validation. With the dataset prepared, the next step was to configure the data augmentation techniques. Eight possible permutations of three techniques were tested: FS, RC, CS, FS+RC, FS+CS, RC+CS, FS+RC+CS, and no data augmentation (Without DAT), and we consider the combination that produces best metric performance as our baseline as for each one. The goal was to identify the best combination of techniques to achieve optimal metrics.

Table 2 presents the mean results for each data augmentation technique, along with the corresponding standard deviation. To evaluate the significance of the differences, a Bonferroni-corrected Wilcoxon signed-rank test was conducted comparing each augmentation condition against the “Without DAT” baseline (N=15 paired observations across 3 feature types and 5 folds, corrected α=0.0071). Significantly improved values are marked with an asterisk (*).

The results reveal that all augmentation combinations except FS alone produce statistically significant improvements over the no-augmentation baseline on the SELD metric, with large effect sizes (rank-biserial r≥0.95). FS applied in isolation actually degrades performance across all metrics (SELD error of 0.368 vs. 0.327 for the baseline), likely because shifting frequency bins without complementary augmentation disrupts spectral patterns that the model relies on, while not providing enough diversity to compensate for this disruption.

Regarding the SED-related metrics, FS+RC+CS achieves the lowest mean ER (0.459) and the highest F1-Score (0.659), outperforming the baseline by 8.38% and 5.78%, respectively. For the DOAE metrics, FS+RC yields the best LE (13.876°), while RC+CS achieves the highest LR (0.686). We note that ER values in the range of 0.46–0.50 are consistent with published results from the DCASE 2021 Challenge and reflect the inherent difficulty of the task, which involves detecting and localizing 12 overlapping sound event classes in reverberant environments.

Considering the composite SELD error, FS+RC+CS obtains the lowest mean score (0.300), representing an 8.25% improvement over the baseline. However, FS+RC, FS+CS, and RC+CS all achieve 0.301, and CS alone reaches 0.302. Pairwise Wilcoxon tests among these top-performing methods yield no significant differences (p>0.15 in all cases), indicating that these configurations are practically equivalent. This suggests that the choice of specific augmentation combination is less important than the decision to apply augmentation at all.

It is worth examining why FS alone performs so poorly while contributing positively in combination. When FS is applied in isolation, it shifts spectral patterns without providing alternative cues for the model to learn from; RC and CS, by contrast, teach the model to cope with missing information and spatial variability, respectively. In combination, FS adds spectral diversity on top of the robustness already provided by RC and CS, yielding a complementary effect. From a practical standpoint, CS alone achieves a 7.6% reduction in SELD error with a single technique—capturing most of the available improvement and offering a favorable trade-off between simplicity and performance.

We also note that the standard deviation for “Without DAT” is lower than for the augmented conditions. This is expected: augmentation introduces stochastic transformations applied per sample and per epoch, which naturally increases fold-to-fold variability. Additionally, the baseline condition produces remarkably consistent mean SELD errors across the three feature types (0.326–0.329), whereas augmentation effects are feature-dependent, inflating the pooled standard deviation.

## 4. Conclusions

In this paper, we investigated various data augmentation techniques to enhance SELD performance. Specifically, we tested combinations of FS, RC, and CS across three different feature sets, i.e., MelSpecIV, LinSpecIV, and SALSA. Our goal was to identify the most effective augmentation strategy for improving both event detection and localization accuracy. The results demonstrate that data augmentation consistently and significantly improves SELD performance: all tested combinations except FS alone reduced the SELD error by approximately 8% compared to training without augmentation, with the differences among effective combinations being statistically indistinguishable. This indicates that the decision to apply augmentation is more consequential than the specific combination chosen. Among single techniques, CS stands out as a particularly efficient option, achieving most of the available improvement with minimal added complexity. We also found that FS alone is detrimental to performance, likely because spectral shifting in isolation disrupts learned frequency patterns without providing compensating diversity; however, FS contributes positively when combined with RC or CS. For future work, we recommend exploring the application of these data augmentation techniques with different model architectures, as our findings are based on a single CRNN architecture. Additionally, investigating other augmentation methods and their interactions, including methods that target spatial diversity beyond channel swapping, could provide further insights into optimizing SELD systems.

## Figures and Tables

**Table 1:** Summary of models and data augmentation techniques used for SELD.

Approach	Model	DA Techniques	$E_{\mathrm{SELD}}$ (Equation (2))
[8]	ResNet-Conformer	ACS+MCS+TDM+TFM	0.17
	ResNet-GRU	ACS+MCS+TDM+TFM	0.18
[9]	CRNN+PKR	F (Frequency Masking)	-
		T (Time Masking)	-
		F-T	-
[10]	CRNN+R.F.	TS	0.57
		PS	0.57
		CR	0.38
[11]	CNN-Conformer	ACS+TFM	0.33
		ACS+TFM+SP	0.33
[12]	SALSA	CS	0.306
		CS+FS	0.255
		CS+FS+RC	0.263

**Table 2:** Performance comparison of data augmentation techniques. Statistical significance assessed via Bonferroni-corrected Wilcoxon signed-rank test against the “Without DAT” baseline (N=15, corrected α=0.0071). Best values per metric are shown in bold.

DAT	SELD	*ER*	F1	*LE*	*LR*
**FS**	0.368 ± 0.049	0.557 ± 0.070	0.566 ± 0.067	17.028 ± 2.412	0.613 ± 0.051
**RC**	0.307 ± 0.022 *	0.469 ± 0.027 *	0.650 ± 0.028 *	14.170 ± 0.970 *	0.671 ± 0.038 *
**CS**	0.302 ± 0.019 *	0.463 ± 0.023 *	0.656 ± 0.024 *	14.306 ± 0.863	0.677 ± 0.031 *
**FS+RC**	0.301 ± 0.020 *	0.461 ± 0.025 *	0.658 ± 0.026 *	**13.876 ± 0.862** *	0.676 ± 0.035 *
**FS+CS**	0.301 ± 0.019 *	0.463 ± 0.027 *	0.656 ± 0.026 *	14.175 ± 0.835 *	0.680 ± 0.028 *
**RC+CS**	0.301 ± 0.019 *	0.465 ± 0.023 *	0.656 ± 0.024 *	14.508 ± 0.989	**0.686 ± 0.033** *
**FS+RC+CS**	**0.300 ± 0.022** *	**0.459 ± 0.029** *	**0.659 ± 0.029** *	14.149 ± 1.031 *	0.680 ± 0.038 *
**Without DAT**	0.327 ± 0.013	0.501 ± 0.016	0.623 ± 0.015	15.115 ± 0.808	0.653 ± 0.031

(*) Denotes a statistically significant improvement over “Without DAT” (Bonferroni-corrected Wilcoxon signed-rank test, p<0.0071, N=15). Rank-biserial effect sizes were large for all significant comparisons (r≥0.95).

## Data Availability

The original contributions presented in this study are included in the article. The source code, built upon the publicly available SALSA framework, will be made available in a public repository upon acceptance. Further inquiries can be directed to the corresponding author.

## References

[1] Tan E.L., Karnapi F.A., Ng L.J., Ooi K., Gan W.S. (2021). Extracting urban sound information for residential areas in smart cities using an end-to-end IoT system. IEEE Internet Things J..

[2] Doell M., Kuehn D., Suessle V., Burnett M.J., Downs C.T., Weinmann A., Hergenroether E. (2024). Automated Bioacoustic Monitoring for South African Bird Species on Unlabeled Data. arXiv.

[3] Suruthhi V., Smita V., Gini J R., Ramachandran K. (2021). Detection and localization of audio event for home surveillance using CRNN. Int. J. Electron. Telecommun..

[4] Chen B., Wang M., Gu Y. (2024). Joint Spatio-Temporal-Frequency Representation Learning for Improved Sound Event Localization and Detection. Sensors.

[5] Chen G., Yu Y., Qiao Y., Yang J., Du C., Qian Z., Huang X. (2024). A Study of Improved Two-Stage Dual-Conv Coordinate Attention Model for Sound Event Detection and Localization. Sensors.

[6] Chen Y., Huang Z., Lei L., Yuan Y. (2025). MSFDnet: A Multi-Scale Feature Dual-Layer Fusion Model for Sound Event Localization and Detection. Sensors.

[7] Ko B.Y., Nam H., Kim S.H., Min D., Choi S.D., Park Y.H. (2022). Data augmentation and squeeze-and-excitation network on multiple dimension for sound event localization and detection in real scenes. arXiv.

[8] Wang Q., Du J., Wu H.X., Pan J., Ma F., Lee C.H. (2023). A four-stage data augmentation approach to resnet-conformer based acoustic modeling for sound event localization and detection. IEEE/ACM Trans. Audio Speech Lang. Process..

[9] Zhang Y., Wang S., Li Z., Guo K., Chen S., Pang Y. (2021). Data Augmentation and Class-Based Ensembled CNN-Conformer Networks for Sound Event Localization and Detection. Technical Report, DCASE 2021 Challenge. https://dcase.community/documents/challenge2021/technical_reports/DCASE2021_Zhang_67_t3.pdf.

[10] Ronchini F., Arteaga D., Pérez-López A. Sound Event Localization and Detection Based on CRNN using Rectangular Filters and Channel Rotation Data Augmentation. Proceedings of the Detection and Classification of Acoustic Scenes and Events 2020 Workshop (DCASE2020).

[11] Zhang J., Ding W., He L. (2019). Data Augmentation and Prior Knowledge-Based Regularization for Sound Event Localization and Detection. DCASE 2019 Detection and Classification of Acoustic Scenes and Events 2019 Challenge. https://dcase.community/documents/challenge2019/technical_reports/DCASE2019_He_97.pdf.

[12] Nguyen T.N.T., Watcharasupat K.N., Nguyen N.K., Jones D.L., Gan W.S. (2022). Salsa: Spatial cue-augmented log-spectrogram features for polyphonic sound event localization and detection. IEEE/ACM Trans. Audio Speech Lang. Process..

[13] Santos O., Rosero K., Masiero B., de Alencar Lotufo R. (2024). w2v-SELD: A Sound Event Localization and Detection Framework for Self-Supervised Spatial Audio Pre-Training. IEEE Access.

[14] Hu J., Cao Y., Wu M., Kong Q., Yang F., Plumbley M.D., Yang J. (2022). A Track-Wise Ensemble Event Independent Network for Polyphonic Sound Event Localization and Detection. arXiv.

[15] Zhong Z., Zheng L., Kang G., Li S., Yang Y. (2017). Random Erasing Data Augmentation. arXiv.

[16] Park D.S., Chan W., Zhang Y., Chiu C.C., Zoph B., Cubuk E.D., Le Q.V. (2019). SpecAugment: A Simple Data Augmentation Method for Automatic Speech Recognition. Proceedings of Interspeech 2019, Graz, Austria, 15–19 September 2019.

[17] Pan Y., Dong F., Yao W., Meng X., Xu Y. (2024). Channel swapping of EEG signals for deep learning-based seizure detection. Electron. Lett..

[18] Kong Q., Cao Y., Iqbal T., Wang Y., Wang W., Plumbley M.D. (2020). PANNs: Large-scale pretrained audio neural networks for audio pattern recognition. IEEE/ACM Trans. Audio Speech Lang. Process..

[19] Nguyen T.N.T., Jones D.L., Watcharasupat K.N., Phan H., Gan W.S. (2022). SALSA-Lite: A fast and effective feature for polyphonic sound event localization and detection with microphone arrays. Proceedings of the ICASSP 2022–2022 IEEE International Conference on Acoustics, Speech and Signal Processing (ICASSP).

[20] Jacome K.G.R., Grijalva F.L., Masiero B.S. (2023). Sound events localization and detection using bio-inspired gammatone filters and temporal convolutional neural networks. IEEE/ACM Trans. Audio Speech Lang. Process..

[21] Mesaros A., Adavanne S., Politis A., Heittola T., Virtanen T. (2019). Joint Measurement of Localization and Detection of Sound Events. Proceedings of the 2019 IEEE Workshop on Applications of Signal Processing to Audio and Acoustics (WASPAA).

[22] Adavanne S., Politis A., Nikunen J., Virtanen T. (2018). Sound Event Localization and Detection of Overlapping Sources Using Convolutional Recurrent Neural Networks. arXiv.

[23] Nguyen T.N.T., Watcharasupat K., Nguyen N.K., Jones D.L., Gan W.S. (2021). DCASE 2021 task 3: Spectrotemporally-aligned features for polyphonic sound event localization and detection. arXiv.

